# Assessing the transmission risk of red sea bream iridovirus (RSIV) in environmental water: insights from fish farms and experimental settings

**DOI:** 10.1128/spectrum.01567-23

**Published:** 2023-09-22

**Authors:** Yasuhiko Kawato, Yuzo Takada, Kaori Mizuno, Shogo Harakawa, Yusaku Yoshihara, Yukihiro Nakagawa, Tomofumi Kurobe, Hidemasa Kawakami, Takafumi Ito

**Affiliations:** 1 Pathology Division, Nansei Field Station, Fisheries Technology Institute, Japan Fisheries Research and Education Agency, Mie, Japan; 2 Ehime Fisheries Research Center, Ehime, Japan; 3 Ainan Town Fisheries Division, Ehime, Japan; 4 Pathology Division, Tamaki Field Station, Fisheries Technology Institute, Japan Fisheries Research and Education Agency, Mie, Japan; University of Prince Edward Island, Charlottestown, Prince Edward Island, Canada

**Keywords:** environmental DNA, eDNA, *Megalocytivirus*, iron flocculation, waterborne virus, *Pagrus major*, IDW, biosecurity

## Abstract

Aquatic animal viruses are considered to be transmitted via environmental water between fish farms. This study aimed to understand the actual transmission risk of red sea bream iridovirus (RSIV) through environmental water among fish farms. An environmental DNA (eDNA) method using iron-based flocculation coupled with large-pore filtration was used to monitor RSIV DNA copies in seawater from fish farms and from an experimental infection model. RSIV dispersion in seawater from a net pen where the disease outbreak occurred was visualized by the inverse distance weighting method using multiple-sampling data sets from a fish farm. The analysis demonstrated that the center of the net pen had a high viral load, and RSIV seemed to be quickly diluted by the tidal current. To evaluate the transmission risk of RSIV in environmental water, the red sea bream *Pagrus major* (approximately 10 g) was exposed to RSIV-contained seawater (10^3^, 10^4^, 10^5^, 10^6^, and 10^7^ copies/L) for 3 days, which mimicked field exposure. A probit analysis of the challenge test indicated that the inferred infection rates of seawater containing 10^5.9^ copies/L and 10^3.1^ copies/L of RSIV were 50% and 0.0001%, respectively. In the surveillance for 3 years at 10 fixed points (*n* = 306), there were only seven samples in which the viral load exceeded 10^4^ copies/L in seawater. These results suggest that the transmission of RSIV among fish farms via seawater is highly associated with the distance between the net pens, and the environmental water is not always an infection source for the transmission of RSIV between fish farms.

**IMPORTANCE:**

Our surveillance of viral loads for red sea bream iridovirus (RSIV) by monitoring environmental DNA in fish farms suggested that the viral loads in the seawater were low, except for the net pens where RSIV outbreaks occurred. Furthermore, our experimental infection model indicated that the infection risk of RSIV-contained seawater with less than 10^3^ copies/L was extremely low. The limited risk of environmental water for transmission of RSIV gives an insight that RSIV could be partly transmitted between fish farms due to the movement of equipment and/or humans from the fish farm where the disease outbreaks. Since our data suggest that seawater can function as a potential wall to reduce the transmission of RSIV, biosecurity management, such as disinfection of equipment associated with fish farms could be effective, even in the semi-open system aquaculture that the environmental water can be freely transferred, to reduce the risk of RSIV outbreaks.

## INTRODUCTION

Red sea bream iridovirus (RSIV) is a double-stranded DNA virus belonging to the genus *Megalocytivirus*, within the family *Iridoviridae* (1). The viral particles with a diameter of 160–180 nm have an envelope-like structure around the icosahedral virion capsid, and their genome size is approximately 110 kbp (2, 3). In the genus *Megalocytivirus*, two species, *infectious spleen and kidney necrosis virus* (*ISKNV*) and *scale drop disease virus* (*SDDV*), are described by the International Committee on Taxonomy of Viruses (ICTV) (4). RSIV is a type of *ISKNV* species that causes mass mortality in cultured fish, with the formation of numerous enlarged cells from which the genus name is derived.

RSIV is the first megalocytivirus (MCV) reported to cause mass mortality in cultured fish. The first case was reported in cultured red sea bream (*Pagrus major*) in Japan in 1990 (5). Since then, viral infection has spread quickly in Japan and has routinely occurred in the summer season in more than 30 mariculture fish species (6, 7). After that, similar MCVs of *ISKNV* species inducing the enlarged cells in the infected cultured fish have been identified in several Asian countries: ISKNV reported from mandarin fish (*Siniperca chuatsi*) in China (8, 9), rock bream iridovirus reported from barred knifejaw (*Oplegnathus fasciatus*) in Korea (10, 11), and turbot reddish body iridovirus reported from turbot (*Scophthalmus maximus*) in China (12, 13). The geographical distribution of MCVs has spread to European countries and the Americas since the 2010s in both food fish (14, 15) and ornamental fish (16, 17). Owing to its significant impact on the aquaculture industry, MCV infection is listed as a notifiable disease by the World Organization for Animal Health as an infection with RSIV (18).

Formalin-inactivated vaccines are commercially available for the management of RSIV outbreaks in fish farms (19, 20), and an RSIV-resistant strain of red sea bream has been developed (21). However, fish farmers do not vaccinate when the vaccine cost is not acceptable compared to the market value of cultured fish. Resistance breeding is not applicable to all RSIV-susceptible species or to wild-caught juveniles in aquaculture. Therefore, appropriate biosecurity management, such as disinfection procedures, could be important in fish farms, as well as vaccination and resistant breeding for integrated pest management of RSIV. On the other hand, RSIV is considered to be horizontally transmitted through environmental water between animals, as immersion challenges have been demonstrated (22). The disinfection procedure might not be effective in transmitting RSIV in a semi-open system culture for marine fish because rearing water (i.e., environmental water) can be freely transferred between net pens. However, the actual transmission risk of RSIV through the environmental water between net pens or fish farms has never been evaluated. Hence, monitoring the appearance or density of RSIV in the aquatic environment surrounding fish farms is important for understanding the RSIV epidemic in fish farms, followed by the implementation of appropriate biosecurity management against RSIV.

Environmental DNA (eDNA) and nucleic acids extracted from environmental samples (water, soil, feces, etc.) have been used extensively since the 2000s, with numerous applications such as metagenomics, species detection, biomass estimation, and monitoring of aquatic animal pathogens (23 – 27). In our previous study (28), we developed an RSIV monitoring method in seawater using an eDNA technique based on iron-based flocculation coupled with large-pore-size filtration (29). In the present study, using the eDNA method, the transmission risk of RSIV in the environmental water was investigated by field sampling in fish farms for 3 years and an experimental challenge test against red sea bream. Based on the sequential data, the actual transmission risk of RSIV in the environmental seawater is discussed in terms of among net pens or fish farms.

## RESULTS

### Seasonal dynamics of RSIV in the seawater around aquaculture environments

**Fig 1:**
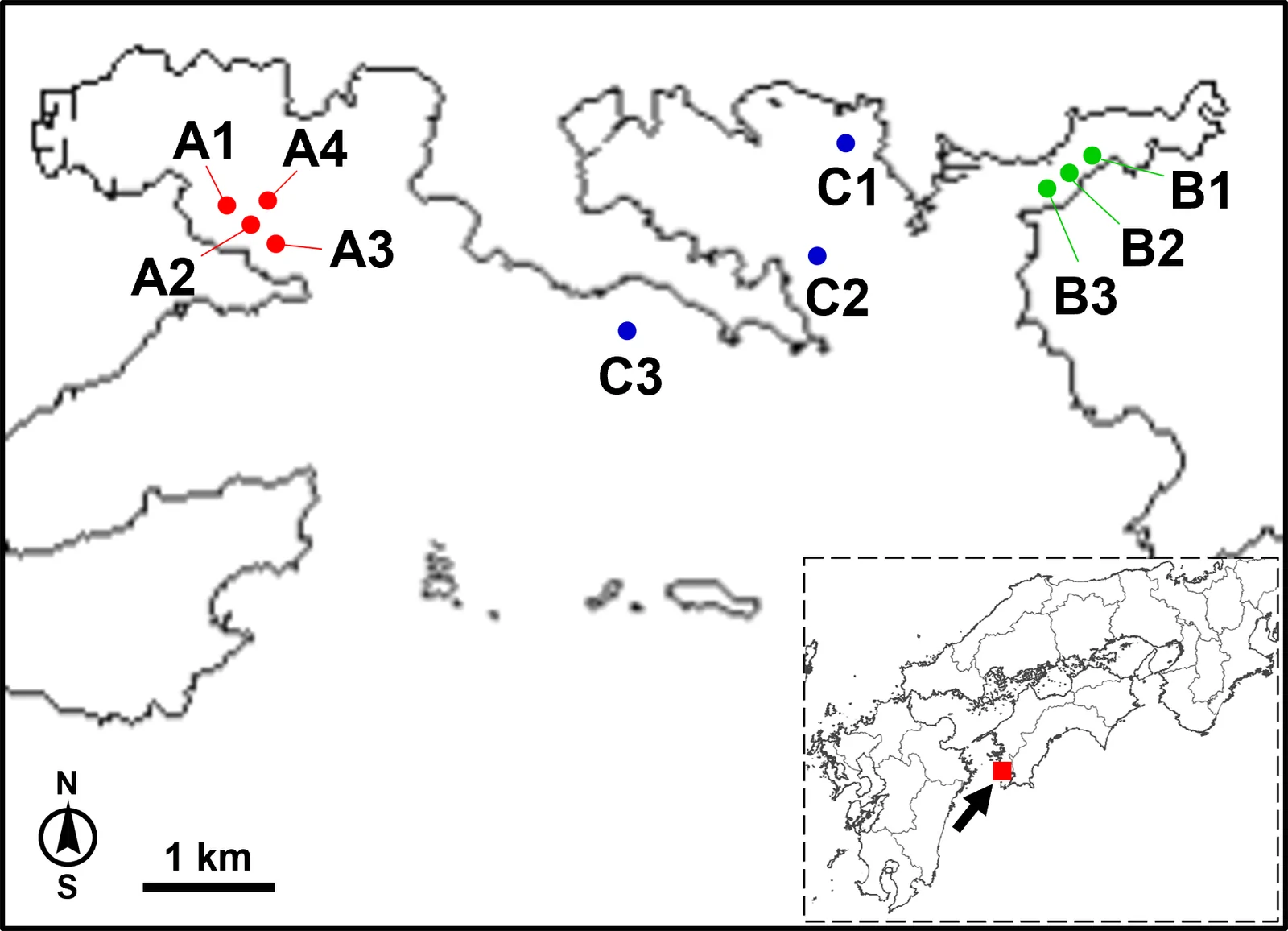
Sampling points of seasonal dynamics of viral load in seawater. The map was edited from a map provided by Geospatial Information Authority of Japan. The red-filled square with an arrow in the wide-area map indicates the sampling area located on Shikoku Island in Japan.

**Fig 2:**
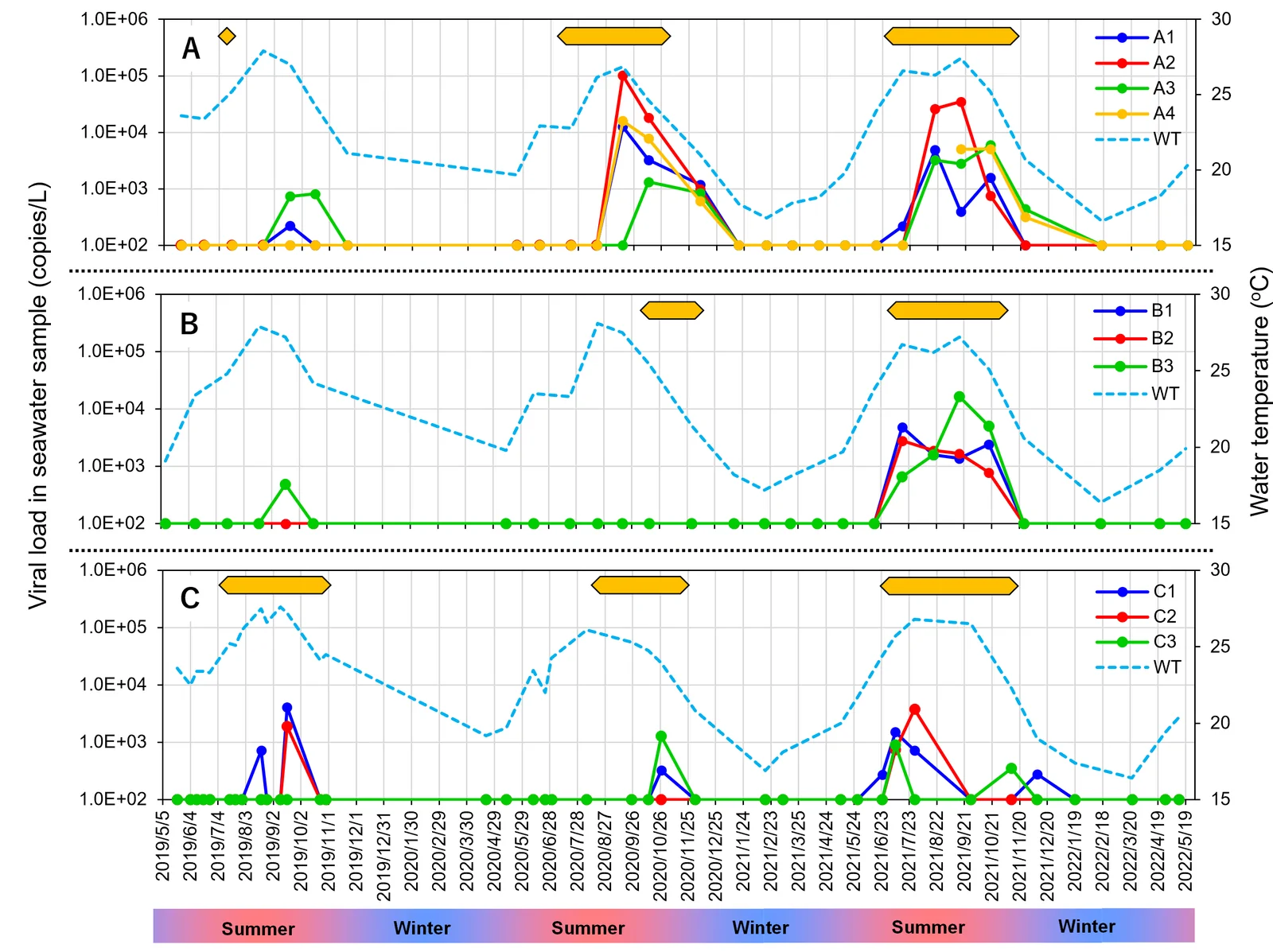
Seasonal dynamics of viral load in seawater from aquaculture environment. Viral load in seawater and water temperature are shown by colored lines and a sky-blue-dashed line, respectively, from area (A, B, and C). The orange-filled belt indicates a term when RSIV outbreak was confirmed in the area based on the information of diagnostic records of RSIV performed by a diagnosis laboratory for fish diseases managed by the local authority. The belt filled with pink and blue under the *X* axis of the graph indicates the schematic season at the sampling area.

Seasonal monitoring of RSIV load in seawater was performed in three culture areas (A, B, and C) using the eDNA method (Fig. 1). A total of 306 seawater samples were analyzed over 3 years between May 2019 and May 2022. During the sampling periods, the total numbers of diagnosed cases of RSIV infection in areas (A, B, and C) were 240, 47, and 89 cases, respectively. The seasonal dynamics of the viral load in seawater are shown in Fig. 2. The limit of detection for RSIV in seawater using the eDNA method is 10^2.0^ copies/L seawater as shown by our previous study (28). RSIV in seawater was detected after the confirmation of the RSIV outbreak in the area by a diagnosis laboratory for fish diseases managed by a local authority (Fig. 2). The maximum viral load was 10^5.0^ copies/L of seawater, which was recorded in area A in September 2020 during the RSIV outbreak at the fish farm where the seawater was collected. RSIV was often detected in seawater during the summer season but disappeared in the winter season (Fig. 2). Indeed, the detection ratio of RSIV from the seawater between July and October when the water temperature exceeded 24°C (33.6%, *n* = 128) was significantly higher than that (6.1%, *n* = 180) between November and June (Fisher’s exact test, *P* value <0.01). RSIV was most frequently detected in 2021 in all sampling areas because of epidemics caused by the introduction of RSIV-infected cultured juveniles. On the other hand, the water depth at each sampling points was not associated with the prevalence or viral load of RSIV. The distribution of viral loads in seawater is summarized in Table 1. The RSIV genome was detected in 54 seawater samples (17.5%) during the 3 years of monitoring. Only seven samples (2.3%) had viral loads exceeding 10^4.0^ copies/L.

**TABLE 1:** Distribution of viral load in seawater for 3 years from aquaculture environment^*a*^

RSIV load (copies/L)	Number of samples in each sampling area			Total (%) *n* = 308
	A (*n* = 111)	B (*n* = 83)	C (*n* = 114)	
<10^2.0^ ^a^	83	70	101	254 (82.4)
10^2.0–3.0^	11	3	8	22 (7.1)
10^3.0–4.0^	11	9	5	25 (8.1)
10^4.0–5.0^	5	1	0	6 (2.0)
>10^5.0^	1	0	0	1 (0.3)

^*a*^ Limit of detection by the eDNA method is 10^2.0^ copies/L seawater (28).

### Visualization of virus dispersion in seawater from net pens during RSIV outbreak

**Fig 3:**
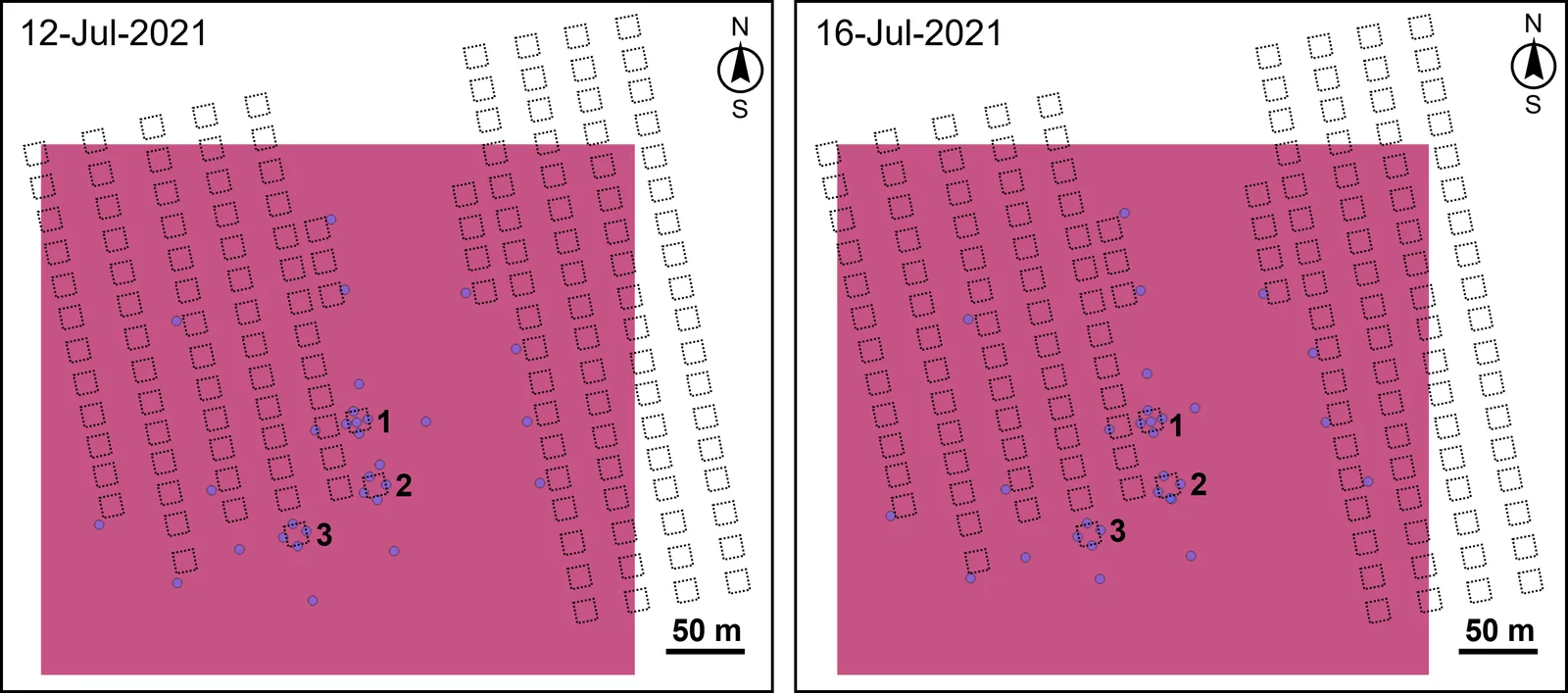
Sampling point for monitoring virus dispersion in a fish farm. Blue dots indicate each sampling point. Dotted squares represent netting pens on fish farms. In net pen 1, water samples were collected from the center and at each edge of a net pen. In net pens 2 and 3, samples were collected only from each edge. The red-filled square shows the analyzed area of the virus dispersion.

**Fig 4:**
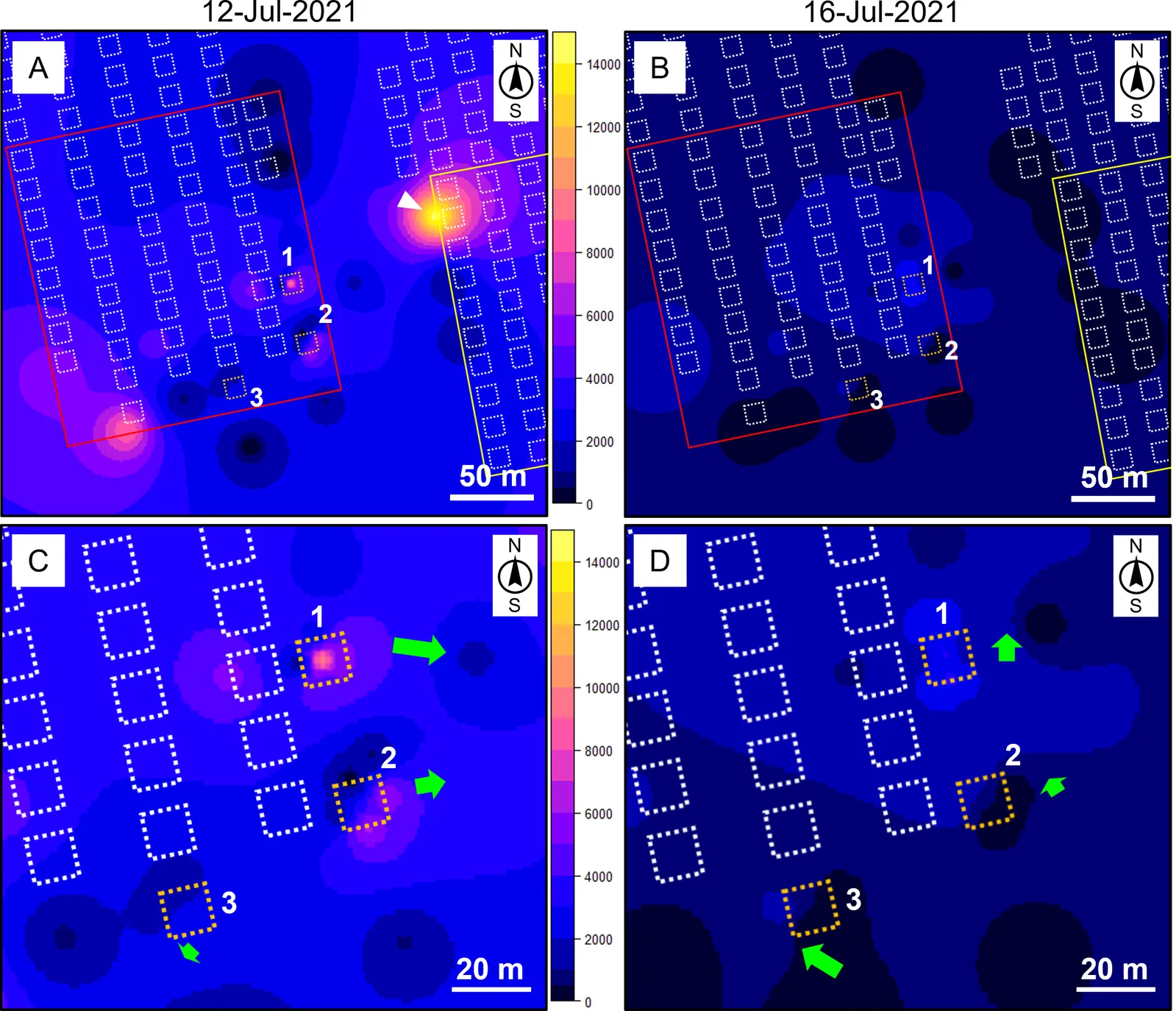
Images of viral dispersion in a fish farm. The viral loads from multiple-sampling points were interpolated by the inverse distance weighting (IDW) method using the gstat package (31). The IDW method can estimate values at unmeasured locations based on the values of nearby measured points, which can be applied for visualizing data by a heatmap (30). (**A**) and (**B**) show the heatmap images of the interpolation assuming the dispersion of RSIV in seawater on 12 July 2021 and 16 July 2021, respectively. Newly introduced juvenile red sea bream were reared in net pens in red-lined areas. Yellow-lined areas indicate net pens in which 1- or 2-year-old red sea bream were reared. The white-filled arrowhead indicates a hot spot observed near the net pens where asymptomatic RSIV-infected fish were presumed to be reared. (**C**) and (**D**) are enlarged images of (**A**) and (**B**), the central parts of each image, respectively. Orange dotted squares with numbers (1, 2, and 3) are net pens from which water samples were collected from the center and/or each edge of the net pens. The green arrows indicate the tidal currents near the net pens. The length and direction of the arrows indicate the schematic speed and vector of the tidal current, respectively.

Two sets of multiple-sampling data sets were obtained from another fish farm during the outbreak of RSIV. The environmental conditions differed between 12 July 2021 and 16 July 2021 (Table 2). The dispersion of RSIV in seawater was visualized using the inverse distance weighting (IDW) method with eDNA data from 30 sampling points on a fish farm (Fig. 3). IDW is an interpolation technique to estimate values at unmeasured locations based on the values of nearby measured points, which can then be used for visualizing data using contour maps or heatmaps (30). The heatmap images shown in Fig. 4 show the inferred RSIV dispersions in seawater at 3 m depth by IDW interpolation between the sampling points. The heatmap image indicates that the viral load in seawater on 12 July 2021 (Fig. 4A) was visually higher than that on 16 July 2021 (Fig. 4B). The mean copy number of the sampling points on 12 July 2021 (10^3.4±0.4^ copies/L) was significantly higher than that on 16 July 2021 (10^2.9±0.4^ copies/L; student’s *t*-test, *P* value <0.01). There was a hot spot (10^4.2^ copies/L) near the net pens rearing 1- or 2-year-old red sea bream (Fig. 4A, arrowhead), which disappeared 4 days later (Fig. 4B). Fig. 4C and D show enlarged images of net pens where seawater sampling was performed at the center and at each edge. On 12 July 2021, RSIV was most abundant at the center of net pen 1 and dispersed toward the right side of the image, probably because of a tidal current toward the right side (Fig. 4C). In contrast, the virus dispersion image seemed to be distorted in net pens 2 and 3, which lacked seawater samples from the center (Fig. 4C). On 16 July 2021, the viral load at the center of net pen 1 was similar to that at the edges of the net pen, suggesting that RSIV was not shed abundantly from the diseased fish in the net pen (Fig. 4D).

**TABLE 2:** Environmental condition for multiple sampling to monitor virus dispersion

Sampling date	Weather	Water temperature^*a*^	Speed of water current^*a*^	Range of tide
12 July 2021	Sunny	24.1 ± 0.2℃	4.0 ± 1.7 cm/s	190 cm
16 July 2021	Cloudy	23.2 ± 0.1℃	2.8 ± 0.1 cm/s	101 cm

^*a*^ Measured at a depth of 3 m.

### Inferring infection risk of seawater containing RSIV

**Fig 5:**
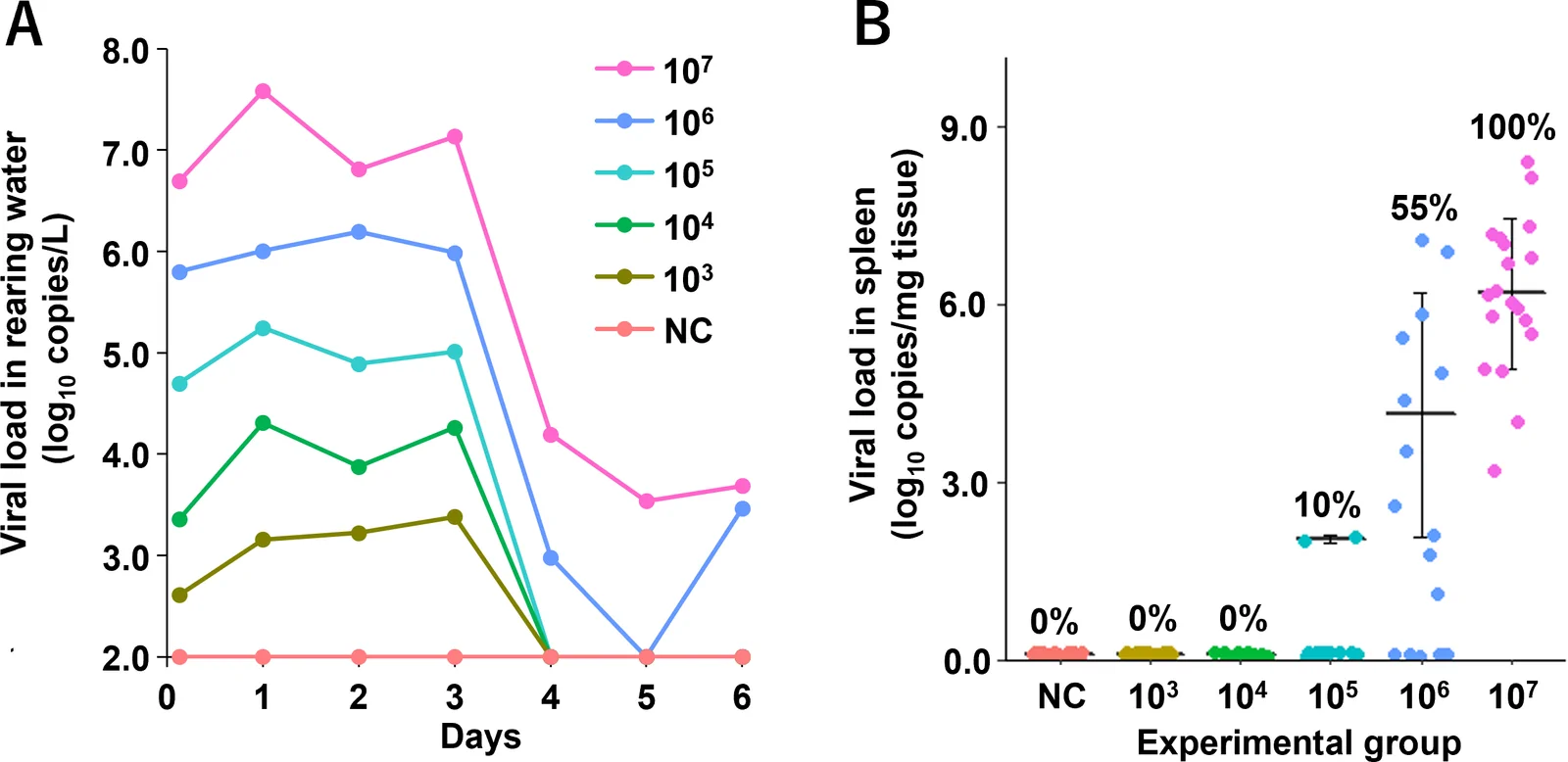
Results of experimental infection that mimics field exposure of RSIV. Seawater containing RSIV ranging between 10^3^ and 10^7^ copies/L was continuously flowed into fish-kept tanks at 1 L/min for 3 days as virus exposure. Then, the fish were reared with the sand-filtrated seawater without virus for 3 days followed by inspection of fish using a real-time PCR assay targeting RSIV. (**A**) Viral load of rearing water during experimental infection. (**B**) Distribution of viral load in the spleen of red sea bream at 6 days post-exposure. The percentage shown above the dot plots indicates the infection rate of each experimental group.

**Fig 6:**
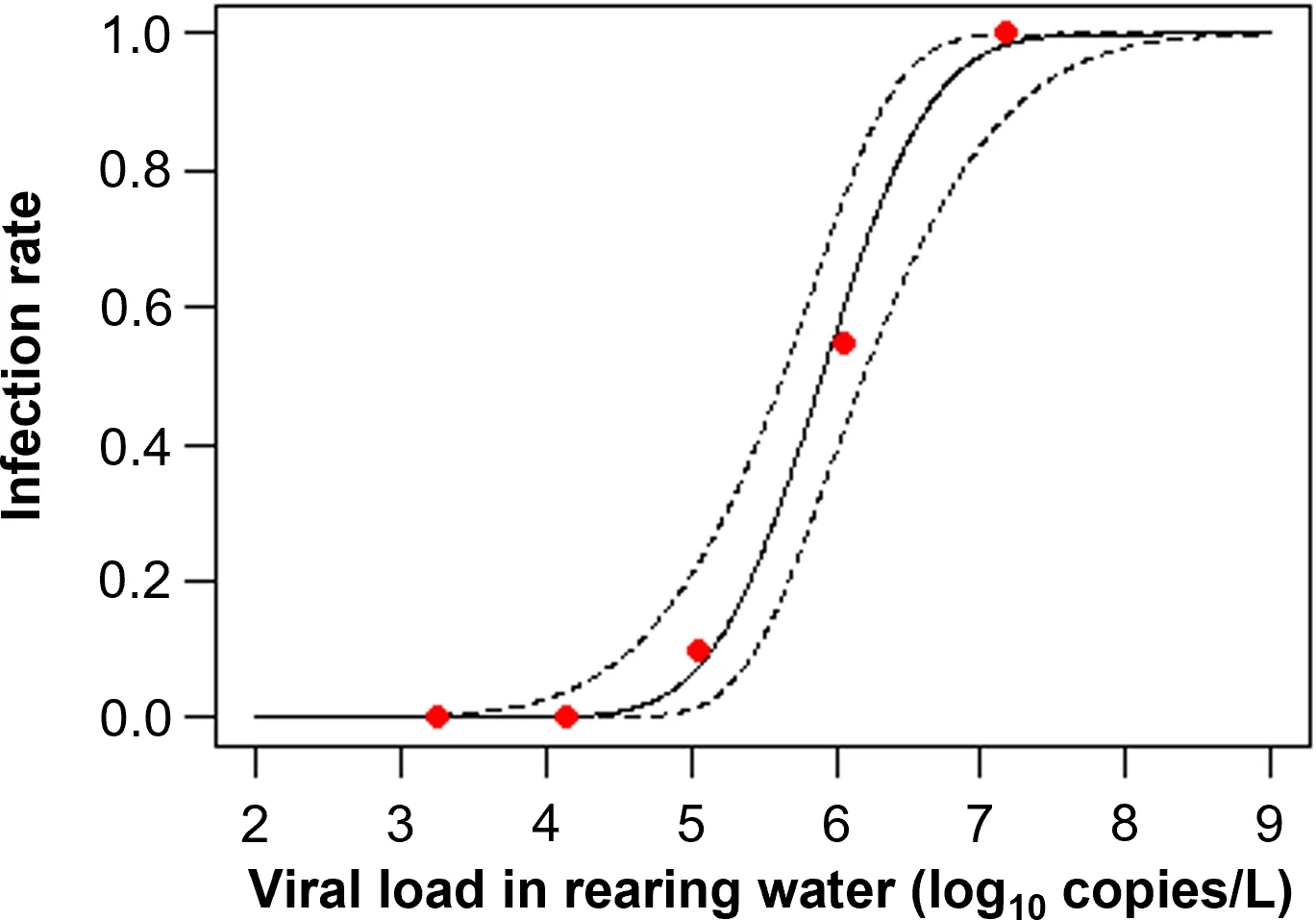
Probit analysis of experimental infection. Black line and dashed line indicate a regression curve by the probit model and 95% CI, respectively. The red-filled dots are the actual infection rate by the experimental infection.

To evaluate the infection risk of RSIV in seawater, red sea bream were exposed via rearing in seawater containing RSIV ranging between 10^3^ and 10^7^ copies/L for three continuous days, which mimics the field exposure of RSIV based on the monitoring data in the aquaculture environment (Fig. S1). The stock of the virus solution supplied to the upper tank was replaced with fresh virus solution daily to ensure the infectious viral titer of RSIV as the infection source. The viral load in the rearing seawater measured by the eDNA method indicated that the fish were exposed to the intended concentration of RSIV for 3 days (Fig. 5A; Table 3). RSIV did not disappear in the experimental groups with 10^6^ and 10^7^ copies/L after stopping the virus supply, indicating that some RSIV-infected fish shed the virus (Fig. 5A). All fish were analyzed 6 days post-exposure to avoid superinfection from infected fish to naïve fish within the tank based on our previous study that viral load in the fish tank was exceeded 10^5^ copies/L at 14 days post-immersion challenge (28). Indeed, the viral loads in rearing seawater of all experimental groups were less than 10^4^ copies/L after stopping the supply of RSIV in this study (Fig. 5A). Although there was one dead fish each in the 10^3^ copies/L and negative control groups, no dead fish were observed in the other tanks during the experiment. The infection rate and viral load of infected fish in each experimental group are shown in Fig. 5B. The infection rates of the 10^5^, 10^6^, and 10^7^ copies/L groups were 10%, 55%, and 100%, respectively, whereas no RSIV was detected in the negative control and 10^3^ and 10^4^ copies/L groups. The mean RSIV genome number in infected fish increased as the exposed viral load increased. The binomial data of infected and non-infected fish in each group were analyzed using a probit regression model (Fig. 6). Using the probit model, the infection rate was inferred using the viral load in seawater as an explanatory variable (Table 4). The infection rate was assumed to be an infection risk for red sea bream when fish were exposed to the viral load via seawater for 3 days. Thus, the infection risk of seawater containing RSIV ranged between 10^3.0^ and 10^4.0^ copies/L, and the mode of the viral load in field monitoring (Table 1) was estimated to be less than 0.1%. Furthermore, seawater containing RSIV at 10^3.0^ copies/L or lower was not found to be a high-risk infection source because the inferred infection risk was less than 0.0001%.

**TABLE 3:** Summary of experimental infection

Experimental group	Mean viral load in seawater for 3 days (log_10_ copies/L)	Infection rate (*n* = 20)	Mean viral load of infected fish (log_10_ copies/mg)
10^7^ copies/L	7.2 ± 0.4	100%	6.2 ± 1.3
10^6^ copies/L	6.1 ± 0.1	55%	4.1 ± 2.1
10^5^ copies/L	5.0 ± 0.2	10%	2.1 ± 0.1
10^4^ copies/L	4.1 ± 0.2	0%	ND
10^3^ copies/L	3.3 ± 0.1	0%	ND
Negative control	ND^*a*^	0%	ND

^*a*^ ND: not detected.

**TABLE 4:** Inferred infection rate by probit analysis

Viral load in seawater^*a*^ (explanatory variable)	Inferred infection rate (response variable)
6.7	90%
5.9	50%
5.1	10%
4.5	1%
4.1	0.1%
3.7	0.01%
3.4	0.001%
3.1	0.0001%

^*a*^ Log_10_ copies/L.

## DISCUSSION

The present study demonstrated the dynamics of RSIV in seawater in an aquaculture environment and estimated the infection risk of seawater containing RSIV based on the viral loads observed in field samples. Few reports have investigated the viral load of rearing water in fish farms, not only for RSIV, but also for other fish viruses. The maximum viral loads recorded in RSIV and salmonid alphavirus in fish farms were 10^6.58^ and 10^5.34^ copies/L seawater, respectively (32, 33). However, there has been no research on aquatic animal pathogens that evaluates the infection risk of rearing water by experimental infection based on the viral load detected in the aquaculture environment. Although the immersion challenge mimics the natural route for fish viruses better than injection, the viral dose and exposure time in previous reports were too high and too short, respectively, to evaluate the actual infection risk (22, 28). In the present study, we selected viral loads ranging between 10^3^ and 10^7^ copies/L, based on the seasonal monitoring. Furthermore, the data from multiple sampling in a fish farm suggested that the viral load in the seawater changed over several days, possibly due to environmental factors such as water temperature and tide current (Fig. 4); hence, fish were exposed to RSIV for 3 days during the experimental infection, assuming that the same viral load does not continue for more than 3 days. The infection rate obtained by the experimental infection was reasonable. Although RSIV-infected fish die within 20 days in an immersion setting (22, 28), actual outbreaks in fish farms are prolonged for more than 40 days (20, 32), indicating that RSIV is gradually transmitted between animals within a net pen. The difference between experimental trials and the transmission situation in the field can be well explained by the inferred infection rate that did not reach 50% when the viral load in seawater was between 10^4.1^ and 10^5.9^ copies/L (Table 4), which was often observed in the net pens with RSIV outbreak (Fig. 2 and 4) (32).

Seasonal monitoring of RSIV using the eDNA method indicated that the viral load in seawater outside net pens in the aquaculture environment was not very high. The viral loads of the RSIV-detected samples were mostly 10^3^ copies/L or lower, although the samples were derived from various situations in terms of geographical locations, water temperatures, water depth, and the frequency of RSIV epidemics. In contrast, images of the RSIV dispersion suggested that viruses shed from RSIV-infected fish were easily diluted and dispersed in seawater (Fig. 4C). Seawater samples for seasonal monitoring were collected from the edges of the net pens, where the viral load was lower than that at the center. Furthermore, the sampling points did not always confirm the RSIV outbreak, even when RSIV was detected from the eDNA, indicating that the detected RSIV genome could be derived from other net pens causing the disease. Therefore, the low viral load in seawater observed in this study seems reasonable. In fact, the range of the RSIV genome number in seawater between 10^2^ and 10^5^ copies/L recorded in this study was similar to the RSIV dynamics in seawater at a fish farm during an RSIV outbreak in other areas (32).

The eDNA method, based on iron-based flocculation coupled with large-pore-size filtration (28, 29), was a useful tool for determining the viral load in seawater samples. Iron flocculation has been used to concentrate various aquatic animal viruses, including piscine nodavirus (34), tilapia lake virus (35), white spot syndrome virus (36), and viral hemorrhagic septicemia virus (37). As shown by various applications, this method allows us to use different but similar viruses with known copy numbers as internal controls, which significantly help in obtaining more accurate viral loads in seawater. In the present study, koi herpesvirus (KHV) was used as an internal control as previously described (32). Using multiplex real-time PCR targeting RSIV and KHV in this study reduced the labor and cost of inspection and increased the reliability of the results by normalizing the copy number of RSIV DNA using the recovery rate of KHV. Indeed, the RSIV dispersion from the net pen was reasonably visualized by considering the tidal current if appropriate sampling points were selected (Fig. 4C). Reasonable visualization of RSIV in seawater is supported by the robust inspection procedure of the eDNA method using an internal control.

We could determine the shedding of RSIV from apparently healthy but RSIV-infected fish using eDNA, despite non-lethal sampling. In this study, a high viral load was observed at a sampling point near the net pen where 1- or 2-year-old red sea bream were reared (Fig. 4A). This fish could be a source of infection for the outbreak of RSIV in juveniles, as shown in a previous study (32). The application of eDNA-based detection of aquatic animal pathogens has been studied for early disease detection (26, 27, 33) or border control (38, 39). The present study also suggests that the eDNA method is an attractive non-lethal tool for the detection of RSIV-infected fish or hotspots of infection on fish farms.

The virus-detected result from the eDNA only indicated the presence of RSIV-shedding fish near the sampling point (assumed to be less than 100 m) and was not representative of the aquaculture environment, such as the entire bay area. eDNA has been used to estimate species or biomass in sampled areas (23, 24) and is a tool that can predict waterborne disease outbreaks in aquatic animals (26, 33, 40, 41). We also expected to be able to detect signs of an RSIV outbreak in a fish farming area using the eDNA method. However, RSIV could not be detected in the seawater before the confirmation of the disease outbreak by the diagnosis laboratory for fish diseases (Fig. 1). The image of the RSIV dispersion indicated that the viral load in the seawater was not identical after 4 days, even in the same fish farm (Fig. 4). In our previous study, increase of RSIV load in eDNA has been observed along with rising water temperature during the disease outbreak in a fish farm (32). We need to note the significance of eDNA data, which could be affected by environmental factors such as water temperature and tidal current, according to the purpose of the research.

Virus distribution and dispersion in the aquaculture environment were successfully visualized using a combination of the eDNA method and IDW interpolation. This study, which has never been attempted in fish pathogens, revealed the actual dynamics of RSIV from a net pen during an RSIV outbreak. However, several limitations of the IDW interpolation were observed. First, the multiple sampling for the analysis required considerable time and labor, as it took approximately 2 h to complete at the 30 sampling points in this study. A longer sampling time is not desirable because environmental factors such as tidal currents can change between the first and last sampling points. Second, the number of sampling points was not sufficient to understand the detailed movement of the RSIV, as several distorted images were observed, such as net pens 2 and 3 (Fig. 4C). Furthermore, although there were hidden hot spots derived from asymptomatic RSIV-infected fish, we could only determine whether the sampling plan was appropriate after completing the analysis (Fig. 4A). In the infectious hematopoietic necrosis virus (IHNV) of Atlantic salmon (*Salmo salar*), finite-volume ocean circulation and particle-tracking models have been applied to simulate virus dispersion via seawater among fish farms (42). The model simulation could be a feasible approach for understanding virus dispersion in fish farms.

Seawater does not threaten the transmission of RSIV between fish farms and even works as a potential wall to reduce the transmission risk of RSIV. Most fish viruses are horizontally transmitted via environmental water between animals within a tank or net pen, as immersion or cohabitation challenges have been demonstrated in fish viruses, including KHV (43), infectious pancreatic necrosis virus (44), RSIV (22), IHNV (45), and piscine nodavirus (34). However, our research suggests that there is a completely different situation between fish farms within a certain physical distance. Based on the experimental infection and probit analyses, more than 50% of the RSIV-detected samples in the seasonal monitoring were not found to be an infection source of RSIV if the cutoff value was set to be 10^3.4^ copies/L, for which the infection risk was estimated to be 0.001%. Indeed, neither a virus nor an outbreak was confirmed at sampling points B1–B3 in 2020, although the RSIV outbreak was confirmed in another fish farm in the area B, which was more than 100 m from the sampling points. Considering the limited infection risk of RSIV-contained seawater, the transmission of RSIV between fish farms could be partly due to the movement of equipment and/or humans associated with fish farms. Therefore, disinfection is considered an important biosecurity management strategy to prevent the invasion of RSIV into fish farms, even in semi-open system cultures in which seawater can be freely transferred between fish farms. Although there have been some reports on the disinfection of MCVs (46, 47), further studies are required to establish appropriate biosecurity management for fish farms in semi-open systems.

### Concluding remarks

The present study, using the eDNA method, demonstrated that the infection risk of seawater containing RSIV is highly associated with viral load, which is affected by the physical distance or tidal currents between fish farms. Viral loads of less than 10^3.4^ copies/L, which are often detected in aquaculture environments, are not a significant source of infection for transmitting RSIV between fish farms. The limited risk of seawater suggests that the disinfection procedure, which has been considered less important in semi-open system cultures, could be essential for biosecurity management to prevent RSIV outbreaks.

## MATERIALS AND METHODS

### Sampling for seasonal monitoring

Three different cultured areas (A, B, and C) located in western Japan were selected for seasonal monitoring of RSIV using eDNA (Fig. 1). Sampling points A1–A4 from area A were net pens derived from a fish farm where red sea bream, white trevally *Pseudocaranx dentex*, and greater amberjack *Seriola dumerili* were cultured. The water depth at A1–A3 and A4 was approximately 20 m and 50 m, respectively. Sampling points B1–B3 from area B were net pens derived from a fish farm where juvenile red sea bream were cultured. Sampling points C1–C3 from area C were net pens derived from three fish farms where the Japanese amberjack, *Seriola quinqueradiata*, was mainly cultured. The water depth at B1–B3 and C1–C3 was approximately 15 m and 50 m, respectively. Information on RSIV outbreaks in each area, including other fish farms in the bay or peninsula area, was obtained from diagnostic records based on the confirmation of enlarged cells using a stamp smear of Giemsa stain (5) inspected by the diagnosis laboratory for fish diseases managed by Ainan town. Sampling was performed once a month from May 2019 to May 2022, while the sampling frequency was reduced in the winter season between December and March when there was no RSIV outbreak. Our preliminary examination indicated that a depth of 3 m, where fish in a net pen are usually swimming, was able to detect the highest copy number of RSIV compared to the surface water at a depth of 1 m or 6 m (data not shown). Seawater was collected from the edge of the net pen at a depth of 3 m using a RIGO-B Transparent water sampler (RIGO Co., Ltd., Tokyo, Japan). The sampled seawater was transferred to Fisheries Technology Institute, Japan Fisheries Research and Education Agency at 4°C and subjected to the iron flocculation method (28) as described within 5 days after sampling. RSIV genome in seawater was stable for at least 1 week when the sampled seawater was kept at 4°C (data not shown).

### Sampling for visualization of virus dispersion

Another fish farm located in Ehime prefecture in western Japan was selected for visualization of virus dispersion in seawater. On the fish farm, red sea bream from yearling to 2-year-old fish were reared, and an RSIV outbreak occurred in the yearling fish in July 2021. Seawater sampling was performed as described above on 12 July and 16 July 2021, at 30 sampling points (Fig. 3). In net pen 1, one sample was collected from the center and at each edge of the net pen. In net pens 2 and 3, samples were collected from each edge only because it was time-consuming to remove the bird netting covering all net pens. At several sampling points where the sampling boat could be anchored with net pens, the water temperature and tidal current (speed and direction) were measured at a depth of 3 m using a handheld 2-D EM current meter with a temperature and depth sensor AEM213-DA (JFE Advantech Co., Ltd.). A laser range finder YARDAGE PRO 500 (Bushnell Co., Ltd. KS, USA) was used to record the locations of the sampling points based on the distance between the nets. Using distance information, the coordinates of the longitude and latitude of the sampling points were determined using an aerial map of the fish farm from Google Maps (https://www.google.co.jp/maps).

### eDNA samples from seawater

The eDNA sample was extracted from 500 mL of seawater using the iron flocculation method coupled with direct DNA extraction from a virus-trapped filter (28, 32). This process was performed at room temperature between 22°C and 26°C. First, approximately 10^3^ copies in 50 µL of heat-inactivated KHV NRIA0301 isolate (48) were added to the seawater sample as an external standard virus for the internal control of eDNA before the iron flocculation process (32). The same volume of KHV in a 1.5-mL microtube was placed at room temperature during the iron flocculation process for each sampling batch as a control for copy number reduction during sample treatment. Then, 50 µL of FeCl_3_ solution (FeCl_3_∙6H_2_O 4.83 g in 100 mL distilled water) was added to the sample seawater so that the final iron concentration was 1 mg Fe/L. The seawater was gently stirred for 1 h using a magnetic stirrer to make the virus flocculate and filtered with a 0.8-µM pore size polycarbonate filter (Advantec Ltd., Tokyo, Japan) under reduced pressure. The flocculate-trapped filter and the external standard KHV were stored in 1.5 mL microtubes at −80°C until eDNA extraction. eDNA was extracted from the flocculate-trapped filter using the DNeasy Blood and Tissue Kit (QIAGEN K.K., Tokyo, Japan), as described previously (32). Since the final DNA elution was performed with 0.2 mL of the elution buffer supplied with the kit, the prepared eDNA was concentrated at 2,500 magnification from the 500 mL sample of seawater.

### Virus preparation

The RSIV RS-17 isolate derived from diseased red sea bream was used (32). The virus was propagated using the SKF-9 cell line (49), derived from spotted knifejaw (*Oplegnathus punctatus*) fry, maintained at 25°C with Hanks’ minimum essential medium (HMEM; GIBCO/Thermo Fisher Scientific K.K., Tokyo, Japan), and supplemented with 10% fetal bovine serum and antibiotic-antimycotic (Gibco/Thermo Fisher Scientific), as described previously (3, 49). RSIV RS-17 was inoculated onto SKF-9 cells seeded in a 75 cm^2^ cell culture flask (Corning, NY, USA) and incubated for 6 days at 25°C. The cell culture medium was then replaced with a fresh medium to increase the final virus production (3). The cell culture supernatant containing RSIV was collected at 17 days post-inoculation and was centrifuged at 2,000 × *g* for 10 min at 4°C. The supernatant was used as the viral solution for the experimental infection. The infectious viral titer (50% tissue culture infectious dose: TCID_50_) of the virus solution was 10^6.8^ TCID_50_/mL, which was determined as previously described (49). The RSIV genome number of the viral solution was determined to be 10^9.7^ copies/mL by DNA extraction using the DNeasy Blood and Tissue Kit and real-time PCR, as described below.

### Experimental infection

Experimental infection of red sea bream was performed following the Animal Research: Reporting of *In Vivo* Experiments (ARRIVE) guidelines. Fish handling, husbandry, and sampling were conducted according to the policies of the Institutional Animal Care and Use Committee of the Fisheries Technology Institute and approved by the committee (IACUC-FTI no. 22009). Red sea bream (approximately 10 g) purchased from a commercial hatchery (A-MARINE KINDAI, Co., Ltd.) was used for experimental infection. They were reared in flowing sand-filtered seawater and fed commercial pellet food OTOHIME EP1 (Marubeni Nisshin Feed Co., Ltd., Tokyo, Japan) daily during the experiment. A total of 120 fish were divided into six groups (i.e., 20 fish per group) and kept in 45 L tanks for 9 days from 24.4°C to 29°C for acclimation of fish. The experimental infection was performed at 28.7°C–29.1°C, which is a relatively high water temperature for red sea bream in order to evaluate a minimum viral load in seawater to establish virus infection under stressful conditions for fish. A schematic of the experimental infection mimicking the natural exposure of the virus to seawater is shown in Fig. S1. Fifteen liters of the virus preparation tank were set on the upper stream of each 45 L tank where 20 fish were kept. Sand-filtered seawater was introduced into the virus preparation tank at a flow rate of 1 L/min. The virus solutions (10^12.7^ copies/L) were diluted into 10^10.7^, 10^9.7^, 10^8.7^, 10^7.7^, and 10^6.7^ copies/L with autoclaved Dulbecco’s phosphate-buffered saline (NISSUI PHARMACEUTICAL Co., Ltd., Tokyo, Japan). They were added to the virus preparation tanks at 0.5 mL/min using peristaltic pumps (Tokyo Garasu Kikai Co., Ltd., Tokyo, Japan) such that the final concentrations of RSIV in each virus preparation tank were theoretically 10^7.4^, 10^6.4^, 10^5.4^, 10^4.4^, and 10^3.4^ copies/L, respectively. Virus-contained seawater flowed into the fish-kept tank at 1 L/min for 3 days for virus exposure. The diluted viral solution was replaced daily with a fresh solution. After 3 days of exposure, the fish were reared in flowing sand-filtered seawater without the virus at 1 L/min for an additional 3 days. For the negative control, fish were reared in flowing sand-filtered seawater at 1 L/min for 6 days. To confirm the viral load in each fish tank, 500 mL of seawater was collected at 3 h and 1, 2, 3, 4, 5, and 6 days post-exposure (dpe), and eDNA was extracted as described above. All the fish were dissected to collect their spleens at 6 dpe. Because one dead fish from the negative control group had no spleen due to cannibalism, the brain was collected for DNA extraction. DNA was extracted from approximately 20 mg of the tissue sample using the Maxwell 16 System DNA Purification Kit (Promega Corporation, Madison, WI, USA). The RSIV genome number in the extracted eDNA or tissue DNA was determined using real-time PCR, as described below.

### Real-time PCR for eDNA

The RSIV genome number in the eDNA samples extracted from seawater was determined using a multiplex real-time PCR assay targeting RSIV (49) and KHV (50). Real-time PCR was used to normalize the RSIV genome number based on the recovery rate of KHV, which was added to the seawater sample as an internal control. The primer/probe set for RSIV consisted of RSIV-MCP186F (5′-CGG CCA GGA GTT TAG TGT GAC T-3′), RSIV-MCP288R (5′-GCT GTT CTC CTT GCT GGA CG-3′), and a hydrolysis probe RSIV-MCP239P (5′-FAM-TGT GGC TGC GTG TTA AGA TCC CCT CCA-BHQ1-3′). The primer/probe set for KHV consisted of KHV-86f (5′-GAC GCC GGA GAC CTT GTG-3′), KHV-163r (5′-CGG GTT CTT ATT TTT GTC CTT GTT-3′), and the hydrolysis probe, KHV-109p (5′-Cy5- CTT CCT CTG CTC GGC GAG CAC G-BHQ2-3′). Each well contained 20 µL of reaction mixture consisting of 2 µL nucleic acid template and 10 µL KAPA PROBE FORCE qPCR Kit (Kapa Biosystems, Inc., Wilmington, MA, Japan), with a final concentration of 200 nM for each RSIV primer and probe, 100 nM for the KHV primers, and 50 nM for the KHV probe. A serial 10-fold dilution of the plasmid containing the target region was used to draw a standard curve for copy number calculations. The real-time PCR assay was performed using the LightCycler 96 Instrument (Roche Diagnostics K.K., Tokyo, Japan) or CFX96 Touch Real-Time PCR Detection System (Bio-Rad Laboratories, Inc., Hercules, CA, USA). The amplification thermal profile consisted of one cycle of 98°C for 3 min and 45 cycles of denaturation and annealing, which were conducted at 95°C for 10 s and 60°C for 30 s. Fluorescence signals for FAM and Cy-5 were recorded in each cycle as RSIV and KHV, respectively. The samples were tested in duplicate. The recovery rate of KHV was calculated using the KHV genome number derived from eDNA samples per KHV DNA (approximately 10^3^ copies), which was added to seawater as the external standard. Based on the recovery rate of KHV, the RSIV genome numbers were normalized and used for data analysis.

### Real-time PCR for fish sample

The RSIV genome number derived from fish samples was determined by real-time PCR using the RSIV-MCP186F/RSIV-MCP288R primers and the RSIV-MCP239P probe, as described above. Twenty microliters of the reaction mixture consisted of 2 µL of the nucleic acid template and 10 µL of THUNDERBIRD Probe qPCR Mix (TOYOBO Co., Ltd., Osaka, Japan), with a final concentration of 200 nM for each primer and probe. A serial 10-fold dilution of the plasmid containing the target region was used to draw a standard curve for copy number calculations. The real-time PCR assay was performed using LightCycler 96 Instrument (Roche Diagnostics). The amplification thermal profile consisted of one cycle of 95°C for 1 min and 45 cycles of denaturation and annealing, which were conducted at 95°C for 10 s and 60°C for 30 s. Samples were tested in duplicate.

### Data analysis

RSIV dispersion in seawater was visualized by interpolation between sampling points by the IDW method (30) using the gstat package (31). Two data sets of viral loads in seawater linked with the coordinates of the sampling points on 12 and 16 July 2021 were used. The samples in which RSIV was not detected were treated as 10^2^ copies/L, which is the limit of detection of the eDNA method, when interpolation of the sampling points was performed using the IDW method. The IDW analysis was performed on R version 4.2.1 using packages “sp,” “rgdal,” “raster,” “leaflet,” “mapview,” and “gstat.”

The relationship between the viral load in seawater and the infection rate obtained by experimental infection was analyzed using a probit regression model. The mean RSIV genome number in the seawater samples from 1 to 3 dpe was used as the viral load in the seawater of each group. On the basis of the mean viral load in seawater and the binomial data of each fish (infection or non-infection), the probit analysis was performed on R version 4.2.1 using “glm” function.

## Data Availability

The data sets generated and/or analyzed during the current study are available from the corresponding author on reasonable request.

## SUPPLEMENTAL MATERIAL

Fig. S1 spectrum.01567-23-s0001.docx Schematic image of experimental infection.

ASM does not own the copyrights to Supplemental Material that may be linked to, or accessed through, an article. The authors have granted ASM a non-exclusive, world-wide license to publish the Supplemental Material files. Please contact the corresponding author directly for reuse.
