# Supplementary material for: Assessing the transmission risk of red sea bream iridovirus (RSIV) in environmental water: insights from fish farms and experimental settings
Source: Microbiol Spectr. 2023 Sep 22;11(5):e01567-23. doi: 10.1128/spectrum.01567-23 (PMC10580957; doi:10.1128/spectrum.01567-23)
Supplement: Fig. S1 — Schematic image of experimental infection. [file spectrum.01567-23-s0001.docx]

**Fig. S1.** Schematic image of experimental infection.

(1): Fifteen liters of the virus preparation tank was set on the upper stream of each 45 L tank where 20 fish were kept. Sand-filtered seawater was introduced into the virus preparation tank at a flow rate of 1 L/min. (2): The virus solutions (10^12.7^ copies/L) were diluted into 10^10.7^, 10^9.7^, 10^8.7^, 10^7.7^, and 10^6.7^ copies/L with autoclaved Dulbecco's phosphate-buffered saline. They were added to the virus preparation tanks at 0.5 mL/min using peristaltic pumps such that the final concentrations of RSIV in each virus preparation tank were theoretically 10^7.4^, 10^6.4^, 10^5.4^, 10^4.4^, and 10^3.4^ copies/L, respectively. (3) and (4): Virus-contained seawater was flowed into the fish-kept tank at 1 L/min for 3 days for virus exposure. (5): After 3 days of exposure, the fish were reared in flowing sand-filtered seawater without the virus at 1 L/min for an additional 3 days. Then, they were dissected to collect their spleens.
